# Preliminary evaluation of palmar tri-radii-related measurements for sex estimation in an Arabian Gulf population sample

**DOI:** 10.1038/s41598-026-59233-z

**Published:** 2026-06-29

**Authors:** Magda Hassan Mabrouk Soffar, Zahraa Khalifa Sobh, Omnia Azzaz, Taqwa Ali Mansoor Abdulla, Mohammed Ibrahim Mahdi Alsaeed, Asmaa Fady Sharif

**Affiliations:** 1 Forensic Medicine and Clinical Toxicology Department, Faculty of Medicine, Alexandria University, Alexandria, Egypt; 2 Medical student at Faculty of Medicine, Alexandria University, Alexandria, Egypt; 3 Forensic Medicine and Clinical Toxicology Department, Faculty of Medicine, Tanta University, Tanta, Egypt; 4 Present Address: Faculty of Medicine, Champollion Street, Alexandria, Egypt

**Keywords:** Palmprint, Palmar tri-radii, Sex estimation, Arabian Gulf population, Anatomy, Health care, Medical research

## Abstract

This cross-sectional study assessed the utility of the distance between palmar tri-radii for sex estimation in an Arabian Gulf population. We enrolled 125 citizens of the Arabian Gulf residing in Alexandria Governorate, Egypt, aged 18–30 years. The palmprints were obtained using fingerprint ink strips. Software (AutoCAD 2025) was used to analyze the 600 dots per inch (dpi)-scanned images of the inked palmprints. We investigated five palmar tri-radii present at the base of the fingers (a, b, c, d) and axial tri-radius ‘t’ situated near the base of the fourth metacarpal. Four distances were measured, including the a-t, b-t, c-t, and d-t distances. Combined abcd-t distance was calculated for each palmprint. With excellent intra- and interobserver agreement, the measured variables exhibited significant sexual dimorphism, with males having larger values. Sex could be predicted from individual measurements, with modest performance. The most significant sex-predictive model incorporated bilateral palmprint measurements: log-odds of being male =-7.710 + 1.074 × (Right d-t) − 0.946 × (Right b-t) + 0.774× (Left a-t) + 0.590 × (Left c-t) − 0.334 × (Left d-t). The model achieved 82.7% sensitivity and an area under the curve of 76.2%. The palmar tri-radii-related measurements could guide sex estimation, alongside other evidence.

## Introduction

Identifying the biological profile, including the sex, remains a critical mission for forensic anthropologists^1,2^. Palmprint is a recognized tool for sex estimation across different populations. A ‘palmprint’ is known as the print of the inner surface of the hand (palm) between the fingers and wrist. Like fingerprints, palmprints possess numerous distinctive features. However, compared with fingerprints, palmprints have a substantially larger surface area. Additionally, creases and principal lines are considered defining characteristics of palmprints^3^.

Approximately 30% of the latent prints recovered at crime scenes are palmprints. There is subsequently an increasing need for palmprint databases worldwide. From a forensic perspective, palmprints are extremely helpful because they include additional details, such as primary lines, minutiae, and tri-radii^4,5^. When other evidence is lacking and print comparisons are not possible because of insufficient comparable data, direct identification cannot be achieved. In these cases, palmprints can still serve as valuable resources for revealing important biological traits of perpetrators, such as sex^6^.

The principal lines divide the palm into three sections: interdigital areas (below the fingers/digits), thenar areas (adjacent to the thumb), and hypothenar areas (toward the little finger or ulnar bone side of the palm). There are typically five tri-radii (sometimes called “delta”) on the palm; four of these are found in the interdigital spaces at the base of each finger, and the axial tri-radius is found close to the wrist. The placement of the tri-radii is considered a crucial landmark for dermatoglyphic analysis of the palmprint^7^. Importantly, palmprint characteristics vary among populations. Therefore, it is necessary to assess the utility of distances between palmar tri-radii in various populations^8^.

Despite these unique features of palm printing and its importance in the forensic field, few studies examining the forensic use of palm tri-radii distances for sex estimation have been published to date, including Egyptian, Sudanese, Indian, Croatian, and Sri Lankan populations^3,7,9–11^. To our knowledge, there are no currently published studies on sex estimation in native Arabian Gulf populations, which are known to share common characteristics. There is a need to establish a reliable method for estimating the sex of the Arabian Gulf population. Therefore, the current study aimed to assess the utility of the distance between the palmar tri-radii as a sex predictor, enabling its use as a tool for establishing a biological profile in a sample of the Arabian Gulf population.

## Subjects and methods

### Study design, and setting

This cross-sectional study included a sample of participating volunteers from the Arabian Gulf area who lived in the Alexandria Governorate, Egypt, during the study period. The recruited sample primarily comprised students enrolled in the Faculty of Medicine at Alexandria University. Others were friends, colleagues in other faculties, and relatives of participating students.

### Sampling and sample size calculation

Convenience sampling was used to reach the largest possible number of participants. The proportion of male subjects in the Arabian Gulf population is assumed to be approximately 0.5. Thus, the minimum sample size should be at least 120, increasing to 125 to account for cases missing one of the measurements. This study included 75 males and 50 females aged 18–30 years, with a mean age of 21.9 ± 2.7 years. There were no significant differences in age between the participating males and females.

### Sample size adequacy

Sample size adequacy was evaluated using the events-per-variable (EPV) heuristic, which recommends a minimum of 10 outcome events per potential predictor^12,13^. At the assumed event rate of 50% and with models presumed to contain two-six predicting variables at maximum, an EPV of 10 corresponds to a required sample size of at least 120 observations. The proposed sample of 125 satisfies this criterion for models retaining up to six predictors and approaches adequacy for larger models. Importantly, all models were estimated using elastic net regularization, which applies a combined L1 and L2 penalty to shrink redundant coefficients toward zero and perform automatic variable selection. This penalization reduces the effective degrees of freedom below the nominal number of entered predictors, relaxing sample size requirements relative to unpenalized logistic regression.

### Inclusion and exclusion criteria

This study enrolled all consenting healthy participants aged between 18 and 30 years. All the participating volunteers were offspring of parents and grandparents from the Arabian Gulf region. Individuals above the age of thirty years were excluded to avoid age-related changes in palmprints, especially the position of the palmar tri-radii^9,14^. Any participant with uncertain or mixed ancestry was excluded. Additionally, participants with recent injuries or dermatological disorders of the hands. Participants with physical abnormalities resulting from burns, fractures, amputations, congenital deformities, degenerative joint diseases or deformities due to a surgical procedure were also not included in our study^3,15^.

### Data collection tool and procedure for tracing palm measurements

For every included participant, demographics, including age and sex, as per the birth proof, were documented. The materials utilized to trace the palmprint included white paper (A4), soap, dry towels, and Fingerprint-Inked Strips 6 × 10 inches (New York, USA), which are made of a thin coating of ink sandwiched between two thin flexible plastic sheets^3,16^. Every participant was instructed to wash both hands with soap and water to obtain the palmprint. The fingerprint ink strips were separated to expose the inked layer. The palm and fingers were rested on these exposed strips, and the back of the whole hand was pressed firmly to flatten the palm, release the creases, and expose the palmar digital triradii that are located at the bases of the fingers. The inked hands were pressed firmly onto white paper, moving from the proximal to the distal, and the palms were then lifted away from the paper, moving in the opposite direction from the distal to the proximal^7,16^. We obtained complete handprints and subsequently cropped them for palmprint analysis.

### Analyzing the palmprint

**Fig. 1:**
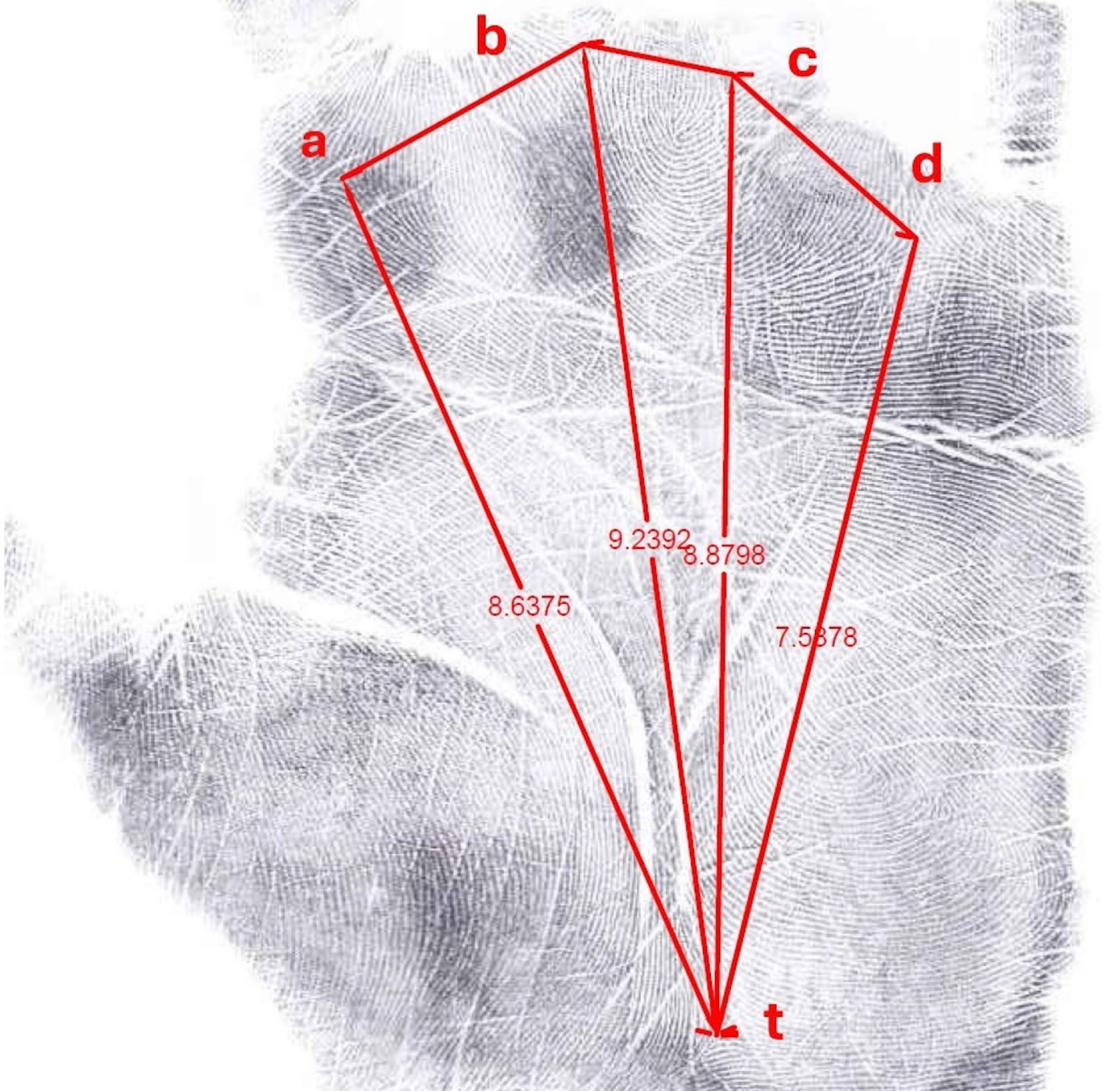
Palmprint showing the five investigated palmar tri-radii namely a, b, c, d and t. All measurements were recorded in cm.

Software (AutoCAD 2025) was used to analyze the 600 dots per inch (dpi)-scanned images of the inked palmprints. We scanned the inked palmprints immediately to avoid any changes due to environmental factors. As Fig. 1 shows, the five Palmar tri-radii on the inked palmprint were identified as follows: Tri-radius ‘a’, which is present at the base of the Index finger; Tri-radius ‘b’, which is located at the base of the middle finger; Tri-radius ‘c’, which is present at the base of the Ring finger; Tri-radius ‘d, which is located at the base of the little finger; and Axial tri-radius ‘t’, which is situated near the proximal end of the palm, the wrist at the base of the fourth metacarpal. A digital ruler was used to measure the distances from the tri-radii a, b, c, and d to the axial tri-radius ‘t’. Four distances were measured in centimeters (cm): a-t, b-t, c-t, and d-t. The combined abcd-t distance was calculated as the arithmetic sum of these four measurements for each palmprint using the formula $\:\mathrm{C}\mathrm{o}\mathrm{m}\mathrm{b}\mathrm{i}\mathrm{n}\mathrm{e}\mathrm{d}\:\mathrm{a}\mathrm{b}\mathrm{c}\mathrm{d}-\mathrm{t}\:=(\mathrm{a}-\mathrm{t})\:+\:(\mathrm{b}-\mathrm{t})\:+\:(\mathrm{c}-\mathrm{t})\:+\:(\mathrm{d}-\mathrm{t}).\:$Additionally, the combined distance for both hands was calculated.

### Reproducibility of the palmprint measurements

The measurements were independently obtained by two investigators to ensure the reliability of the results. Standardized evaluation protocols were applied to reduce measurement error and bias. All measured variables were subsequently traced and reported by each investigator to assess interobserver reliability. Additionally, the first investigator reassessed the measurements after a two-week interval to assess the intra-observer error. Intraclass correlation coefficients (ICCs) were calculated. The data were reported according to the Strengthening the Reporting of Observational Studies in Epidemiology (STROBE) Statement: guidelines for reporting observational studies^17^.

### Data analysis

Analyses were conducted via the R statistical language (version 4.5.0; R Core Team, 2025)^18^. The distribution of continuous numerical variables was examined via the Shapiro‒Wilk test and Q‒Q plots. The variables followed a normal distribution and were summarized using the mean, standard deviation, and range. Comparisons were performed via two-sample t tests (for sex) or paired t tests (for within-sex comparisons of the right and left sides). Univariable logistic regression models were created to predict sex from the measurements. Multivariable models using elastic net regression (which combines LASSO and ridge penalties) were performed to identify relevant predictors while stabilizing estimates. This advanced statistical technique was developed to address limitations inherent in traditional regression approaches, particularly when multicollinearity exists among variables. The elastic net encourages a grouping effect, where strongly correlated predictors tend to be in or out of the model together. The primary advantages of this approach include simultaneous variable selection, coefficient shrinkage to prevent overfitting, improved handling of correlated predictors, and enhanced model stability and predictive accuracy in complex datasets^19^.

Three separate models were constructed: one using right-side measurements, one using left-side measurements, and one using all variables combined. The mixing parameter was set to α = 0.5, equally weighting the LASSO and ridge penalties. The optimal regularization parameter (λ) was selected via 10-fold cross-validation using the cv.glmnet function from the *glmnet* package in R, which minimizes cross-validated binomial deviance. In each fold, the model was trained on 90% of the sample and evaluated on the remaining 10%, repeated across all folds. This internal cross-validation procedure partitions the data into 10 subsets, iteratively training on 9 folds and evaluating on the held-out fold, thereby providing a robust estimate of model generalizability. Receiver operating characteristic (ROC) curve analysis was performed to assess the discriminative performance of the developed regression models. Pairwise comparisons of the area under the curve (AUC) were conducted using bootstrapping and the Z test. A p-value of 0.05 was selected to interpret the results of the statistical tests.

### Ethical approval and consent to participate

Ethical approval was obtained from the Research Ethics Committee of the Faculty of Medicine, Alexandria University, Egypt (IRB Number: 00012098, FWA Number: 00018699, Serial Protocol Number 0307205). The current study followed the World Medical Association Declaration of Helsinki and its subsequent amendments. Informed consent was obtained from all participants prior to their involvement in the research. All data obtained were de-identified by assigning a code number to each case.

## Results

This study enrolled 125 adults from Gulf Arab countries (75 males and 50 females). The participants’ ages ranged from 18 to 30 years, with a mean age of 21.9 ± 2.7 years. There were no significant differences in age between the participating males and females. Repeated measures assessment revealed perfect intra- and interobserver agreement. The intra-observer ICCs ranged between 0.913 and 0.993, whereas the interobserver ICCs ranged between 0.860 and 0.950 (*p* < 0.001), as Table 1 illustrates.

**Table 1:** Inter-observer and intra-observer agreement for the studied palmprint measurements.

Palm measurements	Inter-class correlation (ICC) value	Inter-observer	*p*-value	Intra-observer		
		95% Confidence interval Lower bound, upper bound		Inter-class correlation (ICC) value	95% Confidence interval Lower bound, upper bound	*p*-value
Rt. a-t	0.890	0.850, 0.930	< 0.001*	0.984	0.970, 0.990	< 0.001*
Rt. b-t	0.920	0.900, 0.940	< 0.001*	0.944	0.930, 0.960	< 0.001*
Rt. c-t	0.911	0.890, 0.930	< 0.001*	0.932	0.920, 0.940	< 0.001*
Rt. d-t	0.950	0.940, 0.960	< 0.001*	0.950	0.940, 0.955	< 0.001*
Rt. combined abcd-t	0.885	0.870, 0.900	< 0.001*	0.908	0.895, 0.915	< 0.001*
Lt. a-t	0.907	0.890, 0.920	< 0.001*	0.993	0.990, 0.995	< 0.001*
Lt. b-t	0.913	0.900, 0.920	< 0.001*	0.990	0.985, 0.995	< 0.001*
Lt. c-t	0.900	0.890, 0.910	< 0.001*	0.991	0.990, 0.995	< 0.001*
Lt. d-t	0.892	0.880, 0.900	< 0.001*	0.926	0.920, 0.930	< 0.001*
Lt. combined abcd-t	0.860	0.840, 0.880	< 0.001*	0.913	0.905, 0.920	< 0.001*

ICC values less than 0.5 are indicative of poor reliability, values between 0.5 and 0.75 indicate moderate reliability, values between 0.75 and 0.9 indicate good reliability, and values greater than 0.90 indicate excellent reliability. Rt: right, Lt: left.

a-t – Distance between palmar tri-radius ‘a’ and axial tri-radius ‘t’.

b-t – Distance between palmar tri-radius ‘b’ and axial tri-radius ‘t’.

c-t – Distance between palmar tri-radius ‘c’ and axial tri-radius ‘t’.

d-t – Distance between palmar tri-radius ‘d’ and axial tri-radius ‘t’.

abcd-t distance – arithmetic combined distance of a-t, b-t, c-t, and d-t measurements.

**Fig. 2:**
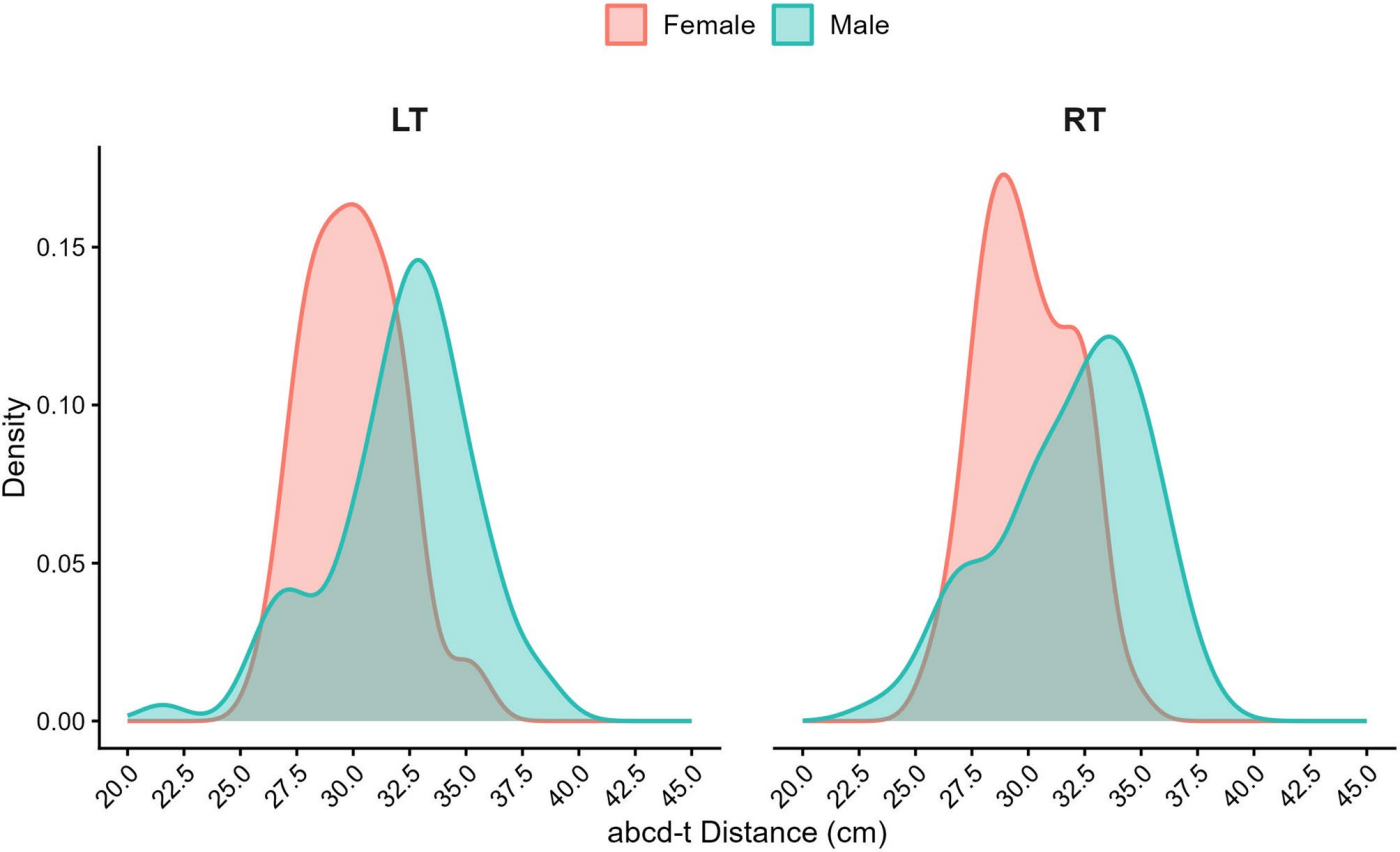
Density plots showing the distribution of abcd-t distances by sex for both palms left (LT) and right (RT).

Figure 2 illustrates the distribution of abcd-t distances by sex for both hands. In both the right and left palmprints, males consistently showed higher abcd-t distances than females. The female distribution was narrower and more peaked, whereas the male distribution was broader and slightly right-skewed, suggesting greater variability in abcd-t distances among males. More than 50% of the males presented combined abcd-t distances ranging between 30 and 34.9, whereas more than 70% of the investigated females presented combined abcd-t distances ranging between 27.5 and 32.4. Table 2 shows that the measured palmprint tri-radii distances (a-t, b-t, c-t, d-t) and the combined abcd-t distance did not significantly differ between right and left hands within the same sex (*p* > 0.05). However, the males had significantly greater measurements (a-t, b-t, c-t, d-t, and the combined abcd-t distance) in both the right and left hands than their female counterparts, with p-values < 0.001 for all 10 measurements.

**Table 2:** Comparison of palmar tri-radii-related measurements in the right and left palmprints between males and female.

Characteristic	Male				Female				Rt. side		Lt. side	
	Rt ^a^ *N* = 75	Lt ^a^ *N* = 75	Cohen’s d (95% CI)	*p*-value ^c^	Rt ^a^ *N* = 50	Lt ^a^ *N* = 50	Cohen’s d (95% CI)	*p*-value ^c^	Cohen’s d (95% CI)	*p*-value ^c^	Cohen’s d (95% CI)	*p*-value ^c^
a-t	8.3 ± 0.7 (6.2–9.6)	8.3 ± 0.7 (5.9–10.0)	0.12 (−0.11 to 0.35)	0.307^*d*^	7.8 ± 0.5 (6.9–8.8)	7.8 ± 0.5 (7.0–9.1)	−0.06 (−0.33 to 0.22)	0.686^*d*^	0.71 (0.34 to 1.14)	< 0.001* ^*e*^	0.81 (0.42 to 1.23)	< 0.001* ^*e*^
b-t	8.5 ± 0.8 (6.3–10.1)	8.6 ± 0.8 (5.8–10.3)	0.24 (0.01 to 0.47)	0.061^*d*^	8.0 ± 0.5 (7.0–9.1)	8.1 ± 0.6 (6.9–9.4)	0.09 (−0.19 to 0.37)	0.517^*d*^	0.65 (0.31 to 1.06)	< 0.001* ^*e*^	0.74 (0.37 to 1.20)	< 0.001* ^*e*^
c-t	8.0 ± 0.9 (5.9–9.7)	8.1 ± 0.9 (5.4–9.8)	0.19 (−0.04 to 0.42)	0.103^*d*^	7.5 ± 0.6 (6.4–9.0)	7.5 ± 0.6 (5.8–9.1)	0.05 (−0.23 to 0.32)	0.737^*d*^	0.71 (0.37 to 1.10)	< 0.001* ^*e*^	0.78 (0.44 to 1.21)	< 0.001* ^*e*^
d-t	7.1 ± 0.9 (4.9–9.0)	7.1 ± 0.8 (4.4–9.1)	−0.03 (−0.26 to 0.19)	0.776^*d*^	6.6 ± 0.5 (5.4–7.8)	6.6 ± 0.5 (5.6–7.9)	0.13 (−0.15 to 0.41)	0.363^*d*^	0.77 (0.40 to 1.20)	< 0.001* ^*e*^	0.70 (0.34 to 1.14)	< 0.001* ^*e*^
Combined abcd-t	31.9 ± 3.2 (23.2–37.8)	32.1 ± 3.2 (21.5–38.8)	0.14 (−0.09 to 0.37)	0.232^*d*^	29.9 ± 2.0 (25.7–34.6)	30.0 ± 2.1 (26.0–35.5)	0.07 (−0.21 to 0.35)	0.627^*d*^	0.73 (0.39 to 1.18)	< 0.001* ^*e*^	0.78 (0.43 to 1.27)	< 0.001* ^*e*^

a Values expressed as Mean ± standard deviation (minimum-maximum), c * Significant at *p* < 0.05, d Paired t test/Paired robust t test, e Welch two-sample t test, Rt: right, Lt: left, CI: confidence interval.

a-t – Distance between palmar tri-radius ‘a’ and axial tri-radius ‘t’.

b-t – Distance between palmar tri-radius ‘b’ and axial tri-radius ‘t’.

c-t – Distance between palmar tri-radius ‘c’ and axial tri-radius ‘t’.

d-t – Distance between palmar tri-radius ‘d’ and axial tri-radius ‘t’.

abcd-t distance – arithmetic combined distance of a-t, b-t, c-t, and d-t measurements.

Table 3 shows that when individual palmar tri-radii measurements were used, sex could be predicted, with accuracies ranging from 64% to 72.8% and modest AUCs of 0.691–0.739. Among the individual predictors, the best observed accuracy was at the left side (a-t) distance, where the a-t distance was greater than 8.15 cm. could predict male sex with an AUC of 0.732, a sensitivity of 68%, and a specificity of 80%. However, there were no significant differences between the AUCs of the investigated measurements. Table 4 presents the 10 proposed sex-predictive models based on individual palmar tri-radii measurements. All the investigated measurements were significant sex predictors (*p* < 0.05). The positive regression coefficients (B) indicate that higher values of the studied parameters were linked to a greater likelihood of being male.

**Table 3:** Receiver operating characteristic (ROC) curve analysis of several palmar tri-radii measurements in the right and left palmprints for the prediction of male sex.

Predictors		AUC (95% CI)	Cutoff	Sensitivity (95% CI)	Specificity (95% CI)	PPV (95% CI)	NPV (95% CI)	Accuracy (95% CI)
The right palmprint								
a-t	0.702 (0.610 to 0.790)		≥ 8.38	52.0 (34.7 to 83.3)	82.0 (54.5 to 98.0)	81.2 (68.8 to 97.0)	53.2 (45.3 to 74.3)	64.0 (58.4 to 77.6)
b-t	0.691 (0.593 to 0.781)		≥ 8.82	42.7 (33.7 to 85.5)	96.0 (54.0 to 100.0)	94.1 (69.6 to 100.0)	52.7 (43.8 to 75.0)	64.0 (57.6 to 76.8)
c-t	0.703 (0.606 to 0.791)		≥ 8.26	45.3 (34.5 to 78.6)	90.0 (65.5 to 100.0)	87.2 (73.5 to 100.0)	52.3 (45.6 to 71.4)	63.2 (58.4 to 76.8)
d-t	0.721 (0.632 to 0.808)		≥ 7.27	50.7 (40.7 to 81.7)	92.0 (62.5 to 98.2)	90.5 (73.9 to 97.8)	55.4 (47.4 to 73.1)	67.2 (60.8 to 78.4)
Combined abcd-t	0.709 (0.617 to 0.792)		≥ 33.12	45.3 (36.4 to 78.7)	98.0 (66.0 to 100.0)	97.1 (76.2 to 100.0)	54.4 (45.7 to 71.0)	66.4 (60.0 to 76.8)
The left palmprint								
a-t	0.732 (0.640 to 0.818)		≥ 8.15	68.0 (49.3 to 80.2)	80.0 (69.1 to 96.2)	83.6 (75.9 to 95.4)	62.5 (50.7 to 74.7)	72.8 (65.6 to 80.8)
b-t	0.720 (0.623 to 0.807)		≥ 8.55	64.0 (43.5 to 79.7)	80.0 (64.6 to 97.8)	82.8 (74.1 to 96.8)	59.7 (48.3 to 72.9)	70.4 (61.6 to 79.2)
c-t	0.729 (0.633 to 0.812)		≥ 8.12	61.3 (47.2 to 84.6)	84.0 (59.6 to 95.6)	85.2 (73.0 to 95.7)	59.2 (48.2 to 75.0)	70.4 (63.2 to 79.2)
d-t	0.723 (0.633 to 0.815)		≥ 7.09	61.3 (50.0 to 76.8)	88.0 (71.4 to 95.7)	88.5 (77.8 to 96.1)	60.3 (49.3 to 72.7)	72.0 (64.8 to 80.0)
Combined abcd-t	0.739 (0.641 to 0.829)		≥ 32.50	53.3 (44.7 to 82.5)	94.0 (66.7 to 100.0)	93.0 (75.3 to 100.0)	57.3 (47.5 to 75.5)	69.6 (63.2 to 80.8)

AUC: area under the curve, CI: confidence interval, NPV: negative predictive value, PPV: positive predictive value, Rt: right, Lt: left.

a-t – Distance between palmar tri-radius ‘a’ and axial tri-radius ‘t’.

b-t – Distance between palmar tri-radius ‘b’ and axial tri-radius ‘t’.

c-t – Distance between palmar tri-radius ‘c’ and axial tri-radius ‘t’.

d-t – Distance between palmar tri-radius ‘d’ and axial tri-radius ‘t’.

abcd-t distance – arithmetic combined distance of a-t, b-t, c-t, and d-t measurements.

**Table 4:** Univariable logistic regression for predicting male sex from palmar tri-radii-related measurements in the right and left palmprints.

Model	Terms	B (95% Confidence interval)	*p* value
The right palmprint			
a-t	Intercept	−7.989 (−13.062 to −3.343)	0.001*
	Right a-t	1.041 (0.465 to 1.673)	0.001*
b-t	Intercept	−6.541 (−11.020 to −2.380)	0.003*
	Right b-t	0.842 (0.338 to 1.386)	0.002*
c-t	Intercept	−6.486 (−10.570 to −2.715)	0.001*
	Right c-t	0.889 (0.403 to 1.418)	0.001*
d-t	Intercept	−6.292 (−10.129 to −2.820)	0.001*
	Right. d-t	0.977 (0.471 to 1.539)	0.000*
Combined abcd-t	Intercept	−7.220 (−11.687 to −3.118)	0.001*
	Right combined abcd-t	0.247 (0.114 to 0.392)	0.000*
The left palmprint			
a-t	Intercept	−9.518 (−15.000 to −4.598)	0.000*
	Left a-t	1.229 (0.620 to 1.912)	0.000*
b-t	Intercept	−7.651 (−12.355 to −3.373)	0.001*
	Left b-t	0.968 (0.455 to 1.535)	0.000*
c-t	Intercept	−7.164 (−11.399 to −3.331)	0.000*
	Left c-t	0.970 (0.480 to 1.514)	0.000*
d-t	Intercept	−6.019 (−10.021 to −2.412)	0.002*
	Left d-t	0.934 (0.411 to 1.517)	0.001*
Combined abcd-t	Intercept	−8.041 (−12.805 to −3.749)	0.000*
	Left combined abcd-t	0.272 (0.134 to 0.426)	0.000*

*Significant at *p* < 0.05, Rt: right, Lt: left.

a-t – Distance between palmar tri-radius ‘a’ and axial tri-radius ‘t’.

b-t – Distance between palmar tri-radius ‘b’ and axial tri-radius ‘t’.

c-t – Distance between palmar tri-radius ‘c’ and axial tri-radius ‘t’.

d-t – Distance between palmar tri-radius ‘d’ and axial tri-radius ‘t’.

abcd-t distance – arithmetic combined distance of a-t, b-t, c-t, and d-t measurements.

Additionally, the palmar tri-radii measurements of the right palmprint, left palmprint, and both hands’ palmprints were combined to develop significant models using elastic net regression with cross-validation, as follows:

$$\:\widehat{log-odds\:of\:being\:male\:}=-6.371\:+\:1.081\times\:(\mathrm{R}\mathrm{i}\mathrm{g}\mathrm{h}\mathrm{t}\:\mathrm{a}-\mathrm{t})\:-\:1.749\times\:(\mathrm{R}\mathrm{i}\mathrm{g}\mathrm{h}\mathrm{t}\:\mathrm{b}-\mathrm{t})\:+\:0.568\times\:(\mathrm{R}\mathrm{i}\mathrm{g}\mathrm{h}\mathrm{t}\:\mathrm{c}-\mathrm{t})\:+\:1.181\times\:(\mathrm{R}\mathrm{i}\mathrm{g}\mathrm{h}\mathrm{t}\:\mathrm{d}-\mathrm{t})$$

I.

$$\:\widehat{log-odds\:of\:being\:male\:}=-7.584\:+\:0.552\times\:(\mathrm{L}\mathrm{e}\mathrm{f}\mathrm{t}\:\mathrm{a}-\mathrm{t})\:+\:0.453\times\:(\mathrm{L}\mathrm{e}\mathrm{f}\mathrm{t}\:\mathrm{c}-\mathrm{t})$$

II.

$$\:\widehat{log-odds\:of\:being\:male\:}=-7.710\:+\:1.074\times\:(\mathrm{R}\mathrm{i}\mathrm{g}\mathrm{h}\mathrm{t}\:\mathrm{d}-\mathrm{t})\:-\:0.946\times\:(\mathrm{R}\mathrm{i}\mathrm{g}\mathrm{h}\mathrm{t}\:\mathrm{b}-\mathrm{t})\:+\:0.774\times\:(\mathrm{L}\mathrm{e}\mathrm{f}\mathrm{t}\:\mathrm{a}-\mathrm{t})\:+\:0.590\times\:(\mathrm{L}\mathrm{e}\mathrm{f}\mathrm{t}\:\mathrm{c}-\mathrm{t})\:-\:0.334\times\:(\mathrm{L}\mathrm{e}\mathrm{f}\mathrm{t}\:\mathrm{d}-\mathrm{t})$$

III. 

The performance of these three models was tested via ROC curve analysis. The AUCs of the models based on separate measurements of the right and left palmprints were 72.7% and 73.5%, respectively. The AUC increased slightly to 76.2% when the model used measurements from both palmprints. However, the combined model achieved the highest accuracy of 70.4%, compared to 65.6% for the model using measurements from the right palmprint and 67.2% for the model using measurements from the left palmprint. The three established models showed good sensitivity of 78.7%, 81.3%, and 82.7%, respectively. The sex-predictive model, which includes tri-radii measurements from both palms, outperforms the other multivariate models in terms of sensitivity, specificity, AUC, and accuracy. However, there were no significant differences in AUCs among the three proposed models, indicating that the forensic utility of the single-hand models is comparable to that of the combined model.

## Discussion

Establishing a biological profile, including sex estimation, is one of the most challenging tasks in forensic practices^1^. The initial step involves analyzing retrieved fingerprints and palmprints. The retrieved fingerprints will be entered into the governmental system for comparison with existing antemortem data^20^. Dermatoglyphics are frequently used to provide evidence about sex^16^. Since palmprints are frequently found at crime scenes, they can be used for estimating sex and assisting in the elimination process by narrowing the pool of suspects during forensic investigations^7^. Specific features of palmprints, such as ridge density and measurements, have been thoroughly examined as indicators of sex^21^.

There is currently limited information on the use of palmar tri-radii (abcd-t) distances as a predictor of sex^9^. The present study aimed to assess the utility of distances between palmar tri-radii (a-t, b-t, c-t, d-t, and combined abcd-t) for sex estimation within a sample of the Arabian Gulf population. The distances from the palmar tri-radii (deltas) ‘a’, ‘b’, ‘c’, and ‘d’ to the axial tri-radii ‘t’ were analyzed individually as well as collectively. Given the significant population growth of the Arabian Gulf region^22^, establishing a reliable database for forensic identification is paramount. The current study provides the first such reference data for the Arabian Gulf population, which forensic practitioners in the region can directly apply to increase the accuracy and reliability of sex estimation from palmprints.

This study revealed that obtaining palmar tri-radii measurements was a reliable tool, as indicated by excellent inter- and intra-observer agreement. The obtained values met the conservative forensic criteria of identification tools proposed by Langley et al.^23^.These findings are consistent with those of previous studies^7,9^. The current study found significant sexual dimorphism across all measured parameters, with males consistently exhibiting longer measurements in both right and left palmprints than females. Table 5 summarizes studies that have investigated sexual dimorphism using palmar tri-radii measurements across different populations. This observation is consistent with the general biological principle of greater soft-tissue dimensions in males, a trend previously reported in various populations, including Egyptians^3^, Indians^7^, and Gujarati populations^24^. However, Jerković et al. investigated different types of tri-radii among the Croatian population, including the distance between four tri-radii located at the base of the fingers. They reported significant sexual dimorphism, with males showing greater measurements than females did in all investigated tri-radii. The proposed univariate sex-predicting models achieved accuracies between 64% and 85%, which increased to 81% and 87%, respectively, with multivariate models, supporting the inclusion of more than a single measurement^9^.

**Table 5:** Literature concerning the use of palmar tri-radii in sex discrimination.

The study	Population	Sample size	Side variations	Right palm						Left palm					
				a-t	b-t	c-t	d-t	abcd-t		a-t	b-t	c-t	d-t	abcd-t	
The current study, 2026	Arabian Gulf populations	125	Insignificant	*	*	*	*	*		*	*	*	*	*	
Banerjee and Das, 2023	Bengali Hindu adults	200	**---**	*	*	*	*	*		*	*	*	*	*	
Jerković et al., 2023**	Croatians (Split and Zagreb)	158	Significant	**A**	**B**	**C**	**D**	**E**	**F**	**A**	**B**	**C**	**D**	**E**	**F**
				*	*	*	*	*	*	*	*	*	*	*	*
Seif et al., 20,222	Egyptians	100	**----**	*	*	*	*	*		*	*	*	*	*	
Badiye et al., 2019	Heterogenous Indians	150	Insignificant	*	*	*	*	**---**		*	*	*	*	**---**	

* Significant variations,.

a-t – Distance between palmar tri-radius ‘a’ and axial tri-radius ‘t’.

b-t – Distance between palmar tri-radius ‘b’ and axial tri-radius ‘t’.

c-t – Distance between palmar tri-radius ‘c’ and axial tri-radius ‘t’.

d-t – Distance between palmar tri-radius ‘d’ and axial tri-radius ‘t’.

abcd-t distance – arithmetic combined distance of a-t, b-t, c-t, and d-t measurements.

** Different types of tri-radii were investigated as follows:.

A) Distance between the tri-radius at the base of the index finger and the tri-radius at the base of the middle finger.

B) Distance between the tri-radius at the base of the middle finger and the tri-radius at the base of the ring finger.

C) Distance between the tri-radius at the base of the ring finger and the tri-radius at the base of the little finger.

D) Distance between the tri-radius at the base of the index finger and the tri-radius at the base of the ring finger.

E) Distance between the tri-radius at the base of the index finger and the tri-radius at the base of the little finger.

F) Distance between the tri-radius at the base of the middle finger and the tri-radius at the base of the little finger.

Indeed, a primary challenge in establishing generalized models for dermatoglyphic sex estimation is the high sensitivity of epidermal ridge configurations to geographic and ancestral lineages. Anthropological literature demonstrates that while sexual dimorphism in palmar dimensions and ridge density remains constant due to underlying biological mechanisms, the absolute baseline measurements fluctuate significantly across distinct ethnic groups^25,26^. Consequently, metric equations or classification baselines developed for specific populations cannot be universally applied without introducing substantial diagnostic error. This high degree of population specificity has historically constrained the global utility of localized dermatoglyphic research, as models optimized for one cohort frequently demonstrate a marked drop in predictive accuracy when validated against external populations^27–29^.

The current findings revealed mean total distance values between abcd-t in the right and left palmprints for males (31.9 and 32.1 cm, respectively) and females (29.9 and 30 cm, respectively). This study also revealed that a combined distance > 33.12 cm on the right and > 32.5 cm on the left increased the likelihood of being male. The obtained values are comparable to those of Badiye et al., who reported that a combined distance of ≤ 30 cm increased the likelihood of being female, whereas values ≥ 32.5 cm were indicative of males^7^. In this study, the highest frequency of combined distances was 30–34.9 in males, whereas it was 27.5–32.4 in females. These observations are consistent with those of Seif et al., who reported that more than 40% of the studied Egyptian males had combined abcd-t distances ranging from 32.5 to 34.9. A similar proportion of Egyptian females presented combined abcd-t distances of 27.5–29.9 and 30–32.4 on the right and left palmprints, respectively^3^. Badiye et al. reported the highest frequency of combined abcd-t distances of 32.5–34.9% in 41.5% and 32.3% of Indian males on the right and left sides, respectively, and 30–32.4% of 33% of females on the right palmprint, while 36.5% of them had combined distances of 27.5–29.9^7^.

In this study, we observed that the a-t distance was the best individual sex predictor and was included in all proposed multivariate models. Banerjee and Das reported similar findings among Bengali Hindu adults. The distance between a and t tri-radius was described as the most significant sex predictor, followed by the combined abcd-t distance, which exhibited a correct sex classification of 80.5 for the right palm. They suggest considering the distance between a and t tri-radius instead of the combined abcd-t distance^10^. Pandey and Vyas investigated the effects of palmar tri-radial angles on the Gujarati population. They reported a direct correlation between the angles adt, atd, and sex and an indirect correlation between sex and the dat angle. These angles are formed by trailing lines drawn from the digital tri-radius (a) to the axial tri-radius (t), from this tri-radius to the digital tri-radius (d), and from the latter to the digital tri-radius (a)^24^.

Another study conducted among Sudanese populations assessed the palmprint ridge density in four areas, including the areas of P3, at the square positioned on the medial mount proximal to the tri-radius of the index finger, with its highest vertex being on the tri-radii of the index, and P4, at another square positioned on the lateral mount proximal to the tri-radius of the little finger, with its highest vertex placed on the tri-radii of the little finger. They described significant bilateral sexual dimorphism in palmprint ridge density, with females having greater ridge density. The maximum potential for sexual estimation was in the right side P4 region and in the left palm P3 area^30^.

The observed sexual dimorphism in the palmar tri-radii is attributed to the common pattern of dimorphism in size and robusticity that results from the interaction of genetic and environmental factors^31^. The observed sex-related variations suggest that the principle of sexual dimorphism in palmar tri-radii distances holds across different ethnic groups and that the specific cutoff values and the most discriminatory individual measurements can vary. These findings highlight the need to establish population-specific standards^9,24^. This study established specific cutoff values for Arabian Gulf populations that could serve as a reference dataset. Future work should also focus on integrating these morphometric data into automated palmprint identification systems to maximize their utility in a practical forensic setting, given that all Arabian Gulf countries use digital dactylography systems to identify admissions^32^.

A strength of this study is the use of advanced statistical modeling, including elastic net regression, which helps to stabilize estimates and select the most relevant predictors in the presence of multicollinearity, thereby improving the generalizability of the models^33^. The proposed multivariate models in this study showed, on average, a fair discrimination power of the proposed models, which is comparable to that of previous studies that focused on palmprint ridge density and described sexing accuracies of 70.2% and 71.8% for the models based on right and left palmprint ridge densities, respectively^21^. Nevertheless, Jerković established multiple models with varying interdigital tri-radii and reported sex prediction accuracies exceeding 80%^9^.

Aligned with the obtained findings, the insignificant side variations in the assessed palmar tr-radii measurable in both sexes have also been reported earlier^7^. However, Jerković et al. reported significant side variations between different tri-radii measurements in the Croatian population^9^. Asymmetrical patterns of body dimensions in the upper limb have been reported in several studies^34,35^. In this study, we observed that measurements on the left palmprint outperformed those on the right palmprint in terms of accuracy (approximately 70%). Additionally, the multivariate model developed from the left palm was slightly better than that developed from the right palm. Compared with the multivariate models developed via both handprint methods, the left a-t and d-t methods achieved comparable or even greater accuracies in sex prediction, which might be beneficial in the case of mutilated bodies in which both complete hands might be unavailable. These findings contradict those of Ali et al., who investigated the effect of sex on palmar ridge density and concluded that the overall accuracy of sex estimation was slightly greater on the right palm^30^. The reported dermatoglyphic asymmetries may be linked to genetic factors, neurological development, or changes in the gestational environment^30^.

One advantage of using palmar tri-radii for sex estimation is their feasibility. At crime scenes, palmprints are often incomplete, making it challenging to obtain full dimensions. Most of the methods described above that use palm prints for identification require a complete print, limiting their effectiveness with partial or lower-quality prints found at the scene. In these cases, the distances between the palmar tri-radii can be used for sex estimation with a high degree of accuracy^9^. Moreover, measuring these distances (abcd-t) is significantly easier than counting palmprint ridge density, which involves complex tasks such as placing and orienting squares and counting ridges, a process that can be difficult and time-consuming^7^.

This study provided a three-tier forensic framework: univariable models for single surviving landmarks, single-hand models for unilateral specimens (one developed model based solely on right palmprint measurements, and another based solely on left palmprint measurements), and the combined model for fully intact palmprints, combining measurements from both hands. This tiered approach was deliberately constructed to reflect real-world forensic scenarios where specimen integrity may be compromised. In cases where only one hand is recoverable or intact, such as in mass disasters, fire victims, or advanced decomposition, the right-hand or left-hand models can be applied independently without any loss of applicability. Importantly, ROC curve analysis demonstrated that the single-hand models perform comparably to the combined model. Therefore, the three introduced models should be used as complementary forensic tools designed for varying degrees of specimen availability. Furthermore, prediction is still feasible using individual palmar triradius measurements, with fair accuracy. This means that even in extreme cases where only a single triradius point is recoverable, sex prediction remains feasible.

A previous study has shown that age can influence the positioning of the tri-radii, which could also potentially affect the proposed methodology^24^. Literature described age-associated changes in skin elasticity and palmar creases, which may affect the visibility of palmprint parameters. The decline in basal keratinocyte proliferation with advancing age leads to thinning of the epidermis, decreased elasticity, and the gradual flattening of epidermal ridges, potentially affecting the clarity of the palmprint and the position of palmar tri-radii^9,14,36^. To avoid age-associated bias, we have chosen an age range of 18–30 years to ensure the full development of sexual dimorphism and to minimize the potential effects of age-related changes on epidermal ridge characteristics, print morphology, and the position of the palmar tri-radii^9,14,16,24^.

Another factor to consider when reporting the palmprint is the adopted methodology and its effect on the quality of the reproduced print. In the current study, we used fingerprint-inked strips to obtain high-quality palmprints, which are much better than those from conventional ink. Fingerprint inked strips usually yield clear, crisp, permanent prints without over-inking due to their thin ink layer. Additionally, these inked strips are washable, non-toxic, and non-allergenic^16,37^. Contact palmprints ensure accurate measurement of linear distances between palmar triradii, as this method captures the palm in its natural position, which maintains the anatomical dimensions among palmar landmarks and minimizes potential distortion^9^. Notably, we avoided the rolling process, traditionally used in fingerprints, as it might introduce stretching and positional displacement of triradii, which would subsequently affect palmar tri-radii distances^14^.

Aside from the incomparable accuracy, the simplicity of the proposed methods, which use multiple tri-radii measurements, compared with the complexity of DNA analysis and the associated costs, is another undeniable advantage^38^. Printing a palm may be an optimal use of resources when DNA is backlogged due to limited corresponding antemortem references^39^. Nevertheless, it is essential to recognize that the characteristics of palmprints, including palmar tri-radii, can be influenced by the quality of the palmprints and their preservation at the crime scene. Additionally, in instances of significant decomposition or severe mutilation, retrieving palmprints becomes unfeasible, necessitating alternative identification methods^38^.

Undeniably, in a field like forensics, the margin of error is critical. No physical measurement can capture a “true value” with absolute certainty, as a margin of error exists across all analytical disciplines. In forensic science, the core responsibility of the practitioner is to isolate known variables, eliminate manageable biases, and statistically estimate the magnitude of any remaining uncertainty^40^. A gray area/margin of error of a proposed model, just like the current study, is questionable. Nevertheless, establishing a consensus error rate for latent print analysis is complicated by legal disputes from opposing parties, meaning no individual study can provide an absolute benchmark^41^. Considering the high specificity and PPV of individual and combined proposed models, these models could determine if predicted sex is truly accurate, which is advisable in forensic practices where false-positive errors are catastrophic and need to be controlled first^40^. Nonetheless, we emphasize that the introduced models cannot be considered conclusive evidence, but only a preliminary screening tool.

## Conclusion

The current study, for the first time, demonstrated that palmar tri-radii measurements could be used to predict sex in the Arabian Gulf population. The modest accuracy reported for the proposed models indicates fair discrimination. However, this level of performance for anthropological parameters is insufficient for standalone forensic identification. Therefore, the present study concludes that measurements of palmar tri-radii can serve as supporting traits for sex estimation when combined with other reliable evidence of sex. Certainly, the findings are preliminary, and their forensic applicability should be for screening rather than definitive identification, to guide legal investigations when other evidence is lacking.

A primary contribution of this work is to provide baseline data for the Arabian Gulf population. Documenting the unique quantitative baselines of the Arabian Gulf population bridges a significant gap in forensic anthropology, transforming a localized population study into a vital comparative reference. Ultimately, this data enables global researchers to map broader macroevolutionary patterns of dermatoglyphic dimorphism and refine multi-ethnic forensic identification frameworks.

## Study limitations

One limitation of the current work arose from the recruitment methods, which undermined the representativeness of the findings obtained. Convenience sampling from a single setting in Egypt limits the generalizability of our findings to the entire population of the Arabian Gulf. Restricting the investigated sample to a specific age range (18–30 years) disregarded the effects of aging, degenerative joint diseases (e.g., arthritis), and loss of skin elasticity on palmar triradius distances. Although we have introduced several predictive models (bilateral and single-hand-based models), a remaining limitation arises in the case of missing hand data, where individual triradius points on a single hand are damaged. Therefore, we recommend future studies with larger samples of diverse ages to explore imputation strategies for partially damaged specimens.

Lack of external validation and the potential for overfitting of the proposed models, despite internal cross-validation, are other limitations, along with the relatively small sample size. We did not employ a fixed training/test split given the modest sample size. Reserving a portion of the sample as a holdout set would substantially reduce the training sample, risking unstable and unreliable parameter estimates. Therefore, the obtained findings should be interpreted with caution. We recommend validating the current conclusions in larger, more diverse, and more representative cohorts across different Arabian Gulf States to strengthen their representativeness and generalizability.

## Data Availability

The datasets used during the current study are available from the corresponding author upon reasonable request.

## Abbreviations

AUC: Area under the curve
cm: Centimeter
DNA: Deoxyribonucleic acid
dpi: Dots per inch
ICCs: Intraclass correlation coefficients
IRB: Institutional review board
ROC: Receiver operating characteristic
STROBE: Strengthening the reporting of observational studies in epidemiology
a-t: Distance between palmar tri–radius ‘a’ and axial tri–radius ‘t’
b-t: Distance between palmar tri–radius ‘b’ and axial tri–radius ‘t’
c-t: Distance between palmar tri–radius ‘c’ and axial tri–radius ‘t’
d-t: Distance between palmar tri–radius ‘d’ and axial tri–radius ‘t’
abcd-t distance: arithmetic combined distance of a–t, b–t, c–t, and d–t measurements
EPV: events-per-variable
